# Oral health condition and development of frailty over a 12-month period in community‐dwelling older adults

**DOI:** 10.1186/s12903-021-01718-6

**Published:** 2021-07-20

**Authors:** Laura Bárbara Velázquez-Olmedo, Socorro Aída Borges-Yáñez, Patricia Andrade Palos, Carmen García-Peña, Luis Miguel Gutiérrez-Robledo, Sergio Sánchez-García

**Affiliations:** 1 Facultad de Odontología, Universidad Nacional Autónoma de México, Ciudad de México, México; 2 División de Estudios de Posgrado e Investigación, Facultad de Odontología, Universidad Nacional Autónoma de México, Ciudad de México, México; 3 Facultad de Psicología, Universidad Nacional Autónoma de México, Ciudad de México, México; 4 Instituto Nacional de Geriatría, Ciudad de México, México; 5 Unidad de Investigación Epidemiológica y en Servicios de Salud, Área Envejecimiento, Centro Médico Nacional Siglo XXI , Instituto Mexicano del Seguro Social, Avenida Cuauhtémoc No. 330, Edificio CORCE, Tercer Piso. Col. Doctores. Alcaldía Cuauhtémoc, 06720 Ciudad de México, México

**Keywords:** Oral health, Frailty, Older adults

## Abstract

**Background:**

To determine the association between oral health condition and development of frailty over a 12-month period in community-dwelling older adults.

**Methods:**

Population-based, case-cohort study derived from the Cohort of Obesity, Sarcopenia, and Frailty of Older Mexican Adults (COSFOMA) study, including data from years 2015 and 2016. Using latent class analysis, we determined the oral health condition of older adults with teeth (t_0_), i.e., functional teeth, presence of coronal caries, root caries, periodontal disease, dental calculus, dental biofilm, root remains, xerostomia, and need for dental prosthesis. Edentulous was considered as a separate class. Criteria of the Frailty Phenotype (t_1_) by Fried et al. were used: weight loss, self-report of exhaustion, walking speed, decreased muscle strength, and low physical activity. The presence of three or more criteria indicated a frail condition. The strength of the association (odds ratio, OR) between oral health condition and development of frailty was estimated through bivariate analysis. Multiple logistic regression was used to adjust for the other variables of study: sociodemographic data (sex, age, marital status, level of education, paid work activity, and living alone), comorbidities, cognitive impairment, depressive symptoms, nutritional status, and use of oral health services.

**Results:**

663 non-frail older adults were evaluated, with a mean age of 68.1 years (SD ± 6.1), of whom 55.7% were women. In t_0_, a three-class model with an acceptable value was obtained (entropy = 0.796). The study participants were classified as: edentulous persons (6.9%); Class 1 = Acceptable oral health (57.9%); Class 2 = Somewhat acceptable oral health (13.9%); and Class 3 = Poor oral health (21.3%). In t_1_, 18.0% (n = 97) of participants developed frailty. Using Acceptable oral health (Class 1) as a reference, we observed that older adults with edentulism (OR 4.1, OR adjusted 2.3) and Poor oral health (OR 2.4, OR adjusted 2.2) were at an increased risk of developing frailty compared to those with Acceptable oral health.

**Conclusion:**

Older adults with edentulism and poor oral health had an increased risk of developing frailty over a 12-month period.

## Background

In recent decades, the population of older adults has increased globally [1] and projections indicate that it will continue to rise; between 2000 and 2050, the proportion of older adults will increase from 11.0 to 22.0%. It is estimated that for the year 2050, the number of dependent older adults will be multiplied by four in developing countries due to physical and mental health problems, as well as frailty [2, 3].

Frailty is one of the most common conditions faced by older adults. It is a physiological state characterized by decreased body capacity and resistance to stressors, resulting from the cumulative decline of multiple physiological systems [4, 5].

Several potential risk factors could be responsible for the development of frailty, such as: Chronic diseases (diabetes, cardiovascular disease, stroke, arthritis, chronic obstructive pulmonary disease, cognitive impairment); physiological factors (active inflammatory processes, anemia, age, immune system dysfunction, endocrine system disruption, underweight or overweight and malnutrition) [6, 7]; sociodemographic and psychological factors (gender, low socioeconomic status, depression, race or ethnicity); and disabilities affecting activities of daily living [6].

Frailty is associated with an increased state of vulnerability and dependence, which increases the risk of adverse outcomes such as falls, delirium and disability [3–5].

Older adults also suffer from poor oral health due to conditions like tooth decay, periodontal disease, and edentulism, among others. These issues accumulate throughout the life course and are exacerbated by infrequent visits to oral health professionals, compared with the general population [8–10].

Oral health deficits can have a biological impact, leading to health problems such as change in salivary flow, altered sense of taste, change in food consumption and inadequate diet, weight loss, low physical function, and presence of chronic inflammation that is caused by poor periodontal status, which in turn is a risk factor for frailty [11–13].

Poor oral health is associated with frailty in older adults [11–14]. The relationship between oral deficits (need for dental prosthesis, number of teeth, periodontal disease, edentulism, oral pain, and chewing capacity) and frailty has been studied in cross-sectional and longitudinal studies [13–17]; however, most studies have considered each of the deficits individually. Two longitudinal studies have investigated this relationship from a holistic approach, considering several oral deficits in order to gain a better understanding of how they interact with frailty [18, 19].

Oral health is not measured directly, but rather is inferred from a set of observed explanatory variables (oral health deficits). Therefore, latent class analysis was used to determine oral health conditions (latent classes) in the sample of older adults. [9, 10]. This study aimed to determine the strength of the association between oral health conditions and development of frailty over a 12-month period in older adults.

## Methods

We conducted a population-based, case-cohort [20] study derived from the Cohort of Obesity, Sarcopenia, and Frailty of Older Mexican Adults (COSFOMA). COSFOMA is a longitudinal study that began in 2014 using a random population sample of 1252 older individuals (60 years and older) who were affiliated with the Mexican Social Security Institute (IMSS). The sampling procedure and the description of the study have been previously documented [21]. The IMSS is part of Mexico's social health protection system; it provides medical services to employees and their families, as well as financial pension benefits for disability, advanced age or retirement. IMSS covers 36.5% of Mexico City's population and approximately 50.9% of older adults [22].

For this study, information from the second (2015) and third wave of data collection (2016) was considered for a 12-month follow-up. We selected the second wave because questions regarding oral health deficits were included that year. The study adhered to national and international ethical guidelines and regulations in research involving human beings, and informed consent was obtained from all participants prior to data collection. This research protocol was approved by IMSS’s National Committee for Scientific Research (Health Research Committee, Ethics Committee on Health Research, and Biosafety Committee on Health Research), registration number: R-2017-785-11.

Using data from the second collection period in 2015 (t_0_), older adults who met two of the five criteria to determine frailty phenotype, according to Fried et al. (Table 1), were selected for the study [5, 21].

**Table 1:** Criteria for frailty phenotype [21]

Weight los	Differences between weight during the previous year and actual weight were calculated. Subjects with weight loss > 10 lb (4.5 kg) during this period were classified as positive for the criterion of weight loss
Self-report of exhaustion	Two questions were used from the revised version of 35 items of the Center for Epidemiologic Studies Depression Scale (CESD-R) adapted for older Mexican adults to determine the criteria of exhaustion. Items considered were: “I felt that everything I did was with difficulty” and “I could not continue.” Considered positive for the criteria was if the participant responded: “During 5–7 days in the past week” or “Almost every day for 2 weeks”
Low physical activity	Level of physical activity during the previous week was evaluated with the Physical Activity Scale for the Elderly (PASE) questionnaire that included self-reported occupational, domestic, and recreational activities. Low physical activity was considered as ≤ 58.6 points for men and ≤ 56.4 points for women (low point quartile of PASE)
Slowness	Walking time was estimated for 4.5 m (15 ft), stratified by sex and stature. Walking distance was considered in women with a height ≤ 159 cm, time ≥ 7 s and height > 159 cm, time ≥ 6 s. Walking distance was considered in men with a height ≤ 173 cm, time ≥ 7 s and with a height < 173 cm, time ≥ 6 s
Weakness (low grip strength)	Grip strength of the nondominant hand was evaluated using dynamometry (Takei T.K.K5001, Takei Scientific Instruments Co. Ltd., Tokyo, Japan) with values stratified by sex and BMI quartiles. In women, low grip strength was considered with BMI ≤ 23.0, ≤ 17 kg; BMI 23.1–26.0, ≤ 17.3 kg; BMI 26.1–29.0, ≤ 18.0 kg; BMI > 29, ≤ 21.0 kg. In men, it was considered with BMI ≤ 24.0, ≤ 29 kg; BMI 24.1–26.0, ≤ 30.0 kg; BMI 26.1–28.0, ≤ 30 kg; BMI > 28, ≤ 32.0 kg

**Fig. 1:**
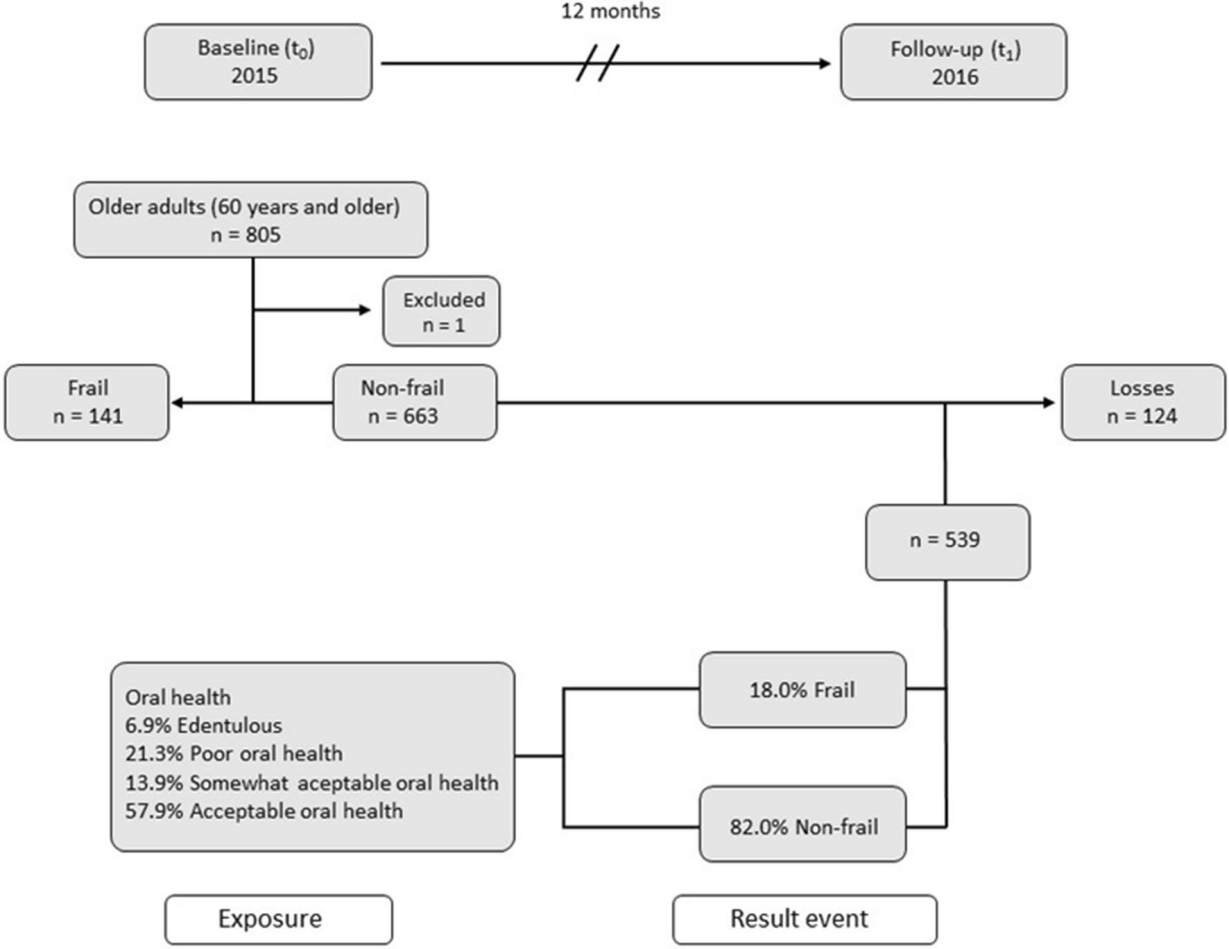
Diagram of the case-cohort study: oral health condition associated with the development of frailty over a 12-month period in community‐dwelling older adults

A total of 663 non-frail older adults were identified. Those with missing information and who did not attend follow-up or died were excluded. The final sample comprised 539 older adults in the 12-month follow-up period (Fig. 1). Invitation letters were sent to the homes of the selected individuals, specifying the date and time that they should attend the XXI Century National Medical Center in Mexico City. A phone number was provided for those who needed to change their appointments. Subsequently, they received a telephone call to remind them about their appointment and new schedules were organized for those who could not attend as planned.

Data on oral deficits were obtained through a clinical dental evaluation by two oral health professionals after previous standardization of procedures (Kappa ≥ 0.80). Disposable gloves, front lamp, mirror number 5 (front reflection), and CPI probe (WHO style-probe (CP-11.5B)) were used. The examination included presence of: Functional teeth (< 20 teeth, ≥ 20 teeth); coronal caries; root caries (presence/absence); periodontal disease (presence/absence of clinical attachment, ≥ 4 mm in at least one tooth); dental calculus (presence/absence); dental biofilm (presence/absence); root remains (presence/absence) [23]; xerostomia, for which the Xerostomia Inventory (≥ 28 points) was used [24]; need for removable dental prosthesis (absence of stability and retention) [25]; and the presence or absence of edentulism (total absence of teeth in the oral cavity). These oral conditions were used to perform latent class analysis, categorizing oral health in older adults; edentulism was not included because it is an independent category.

Other variables used in the study were sociodemographic characteristics (sex, age, marital status, level of education, paid work activity, and living alone). For comorbidities (the information was self-reported), data from the Elixhauser Index was taken as a reference [26]. Cognitive function was evaluated using the Mini-Mental State Examination (MMSE); a score ≤ 23 was considered as cognitive impairment, adjusted for education level [27]. Depression was assessed through the Center for Epidemiologic Studies Depression Scale-Revised (CESD-R); a score of ≥ 57 points indicates presence of depressive symptoms [28]. Finally, nutritional status was evaluated using the Mini Nutritional Assessment (MNA), in which < 17 points indicate malnutrition; the categories "risk of malnutrition and satisfactory nutrition" were combined into a single variable [29]. Use of dental services (self-reported) in the past 12-months was recorded (Yes/No).

To determine the frailty phenotype after 12-months (t_1_), the database from the third data collection period, spanning April to September 2016, was used. The following criteria were considered: weight loss, self-report of exhaustion, low level of physical activity, slow walking speed, and low muscle strength (Table 1). For this study, frailty in older adults was defined as meeting three or more of the above criteria for both baseline and follow-up [5, 18].

### Statistical analysis

Descriptive statistics were used to obtain the frequency, mean (Standard Deviation, SD), and median (percentile 25–75) of the variables that were considered at the start of tracking (t_0_). For high coronal caries, greater than or equal to 2 decayed teeth (75th percentile value) was considered as a cutoff point.

To categorize oral health in older adults at the onset of a 12-month follow-up, a Latent Class Analysis (LCA) was performed. The presence of < 20 functional teeth, coronal caries (≥ 2), root caries, periodontal disease, dental calculus, dental biofilm, root remains, xerostomia, and need of dental prosthesis was considered for this analysis. Latent Class Analysis (LCA) is a person-centered approach that aims to classify individuals into mutually exclusive groups by building oral health categories that are based on oral health deficits [9, 10].

In this study, LCA was used to create an optimal number of classes to categorize oral health in older adults [10]. The choice of the model, in terms of the number of oral health classes, was determined by progressively increasing the number and contrasting the results with those of each subsequent model using the Lo-Mendell-Rubin (LMR) likelihood ratio test of model fit; in the LMR, the presence of a non-significant probability (*p* > 0.05) suggests that the previous model (with fewer classes) is preferable [30, 31].

A combination of the Akaike Information Criterion (AIC) and the Bayesian Information Criterion (BIC) [32, 33] was used to evaluate the goodness of fit of the model, as well as the entropy values [30]. Although there is no standard threshold for evaluating entropy, values close to 1.0 are the most desirable [9, 10, 32]. Finally, the models were interpreted in terms of their theoretical and practical consistency. The most simple and parsimonious model was selected, using the software Mplus, version 7.0.

The strength of the association (odds ratio, OR and 95% confidence interval, 95% CI) between oral health in older adults and the development of frailty over a period of 12-months was estimated. Bivariate and multiple logistic regression was used to adjust for the other variables of study: sociodemographic data (sex, age, marital status, level of education, paid work activity, and living alone), comorbidities, cognitive impairment, depressive symptoms, nutritional status, and use of oral health services. The group of non-frail older adults was taken as a reference. The IBM-SPSS statistical package for Windows, Version 23.0 (IBM Corp. Released 2015, Armonk, NY: IBM Corp.) was used to perform the analyses.

## Results

The baseline assessment (t_0_) was carried out in 2015. A total of 805 older adults were included in the study, with a mean (SD) age of 69.0 (6.7) years. Of these, 58.5% (n = 471) were women and 41.5% (n = 334) men, with a mean age of 69.2 (6.5) and 68.9 (6.8) years, respectively. Frailty was found in 17.5% (n = 141) and non-frailty in 82.5% (n = 664) of participants. After exclusion of 141 frail persons in T_0_ and one person who rejected oral examination, the final population was 663 older adults. The mean age was 68.1 (6.1); 55.7% (n = 369) were women and 44.3% (n = 294) men, with a mean age of 67.7 (6.0) and 68.7 (6.2), respectively.

For the 12-month follow-up, 81.3% (n = 539) of participants were available. The mean age was 67.9 (6.0); 55.1% (n = 297) were women and 44.9% (n = 242) men, with a mean age of 67.4 (5.8) and 68.6 (6.1), respectively. Furthermore, 18.7% (n = 124) of participants were lost to follow-up, where: 5.7% (n = 7) died, 4.8% (n = 6) were sick at home; 31.5% (n = 39) refused to participate; 48.3% (n = 60) could not be located; 5.7% (n = 7) did not have someone to accompany them to the study; 2.4% (n = 3) had some disability; and 1.6% (n = 2) worked, so they were not able to attend their appointment (Fig. 1).

Table 2 shows baseline characteristics according to frailty condition in follow-up; 18.0% (n = 97) of older adults developed frailty over a period of 12-months (2015–2016). A heterogeneous distribution according to frailty and age was observed. In addition, 62.0% (n = 362) reported being married or in a free union, 40.1% (n = 216) performed some paid work activity, and 3.5% (n = 19) had depressive symptoms, while 63.6% (n = 343) reported not having used oral health services in the past 12-months, with heterogeneity observed for frailty. Non-significant differences were noted in the remaining characteristics.

**Table 2:** Basal characteristics of the study sample of older adults

	Total	Non-frail	Frail	*p*-value*
	% (n)	% (n)	% (n)	
	100 (539)	82.0 (442)	18.0 (97)	
*Sex*				
Men	44.9 (242)	46.2 (204)	39.2 (38)	.211
Women	55.1 (297)	53.8 (238)	60.8 (59)	
*Age (years)*				
≥ 80	5.8 (31)	4.1 (18)	13.4 (13)	< .001
70–79	27.0 (146)	25.3 (112)	35.1 (34)	
60–69	67.2 (362)	70.6 (312)	51.5 (50)	
*Marital status*				
Widowed	18.2 (98)	18.3 (81)	17.5 (17)	.743
Single	19.8 (107)	19.2 (85)	22.7 (22)	
Married/free union	62.0 (334)	62.4 (276)	59.8 (58)	
*Education level*				
None	3.0 (16)	3.2 (14)	2.1 (2)	.093
1–6 years	25.4 (137)	23.5 (104)	34.0 (33)	
≥ 7 years	71.6 (386)	73.3 (324)	63.9 (62)	
*Salaried work*				
No	59.9 (323)	59.3 (262)	62.9 (61)	.511
Yes	40.1 (216)	40.7 (180)	37.1 (36)	
*Living alone*				
Yes	12.1 (65)	12.0 (53)	12.4 (12)	.917
No	87.9 (474)	88.0 (389)	87.6 (85)	
*Comorbidities*				
≥ 2	18.6 (100)	18.3 (81)	19.6 (19)	.541
1	27.1 (146)	26.3 (116)	30.9 (30)	
0	54.3 (293)	55.4 (245)	49.5 (48)	
*Cognitive impairment*				
Yes	17.4 (94)	16.1 (71)	23.7 (23)	.072
No	82.6 (445)	83.9 (371)	76.3 (74)	
*Depression*				
Yes	3.5 (19)	2.0 (9)	10.3 (10)	< .001
No	96.5 (520)	98.0 (433)	89.7 (87)	
*Malnutrition*				
Yes	2.8 (15)	2.7 (12)	3.1 (3)	.838
No	97.2 (524)	97.3 (430)	96.9 (94)	
*Use of oral health care services in the past 12-months*				
Yes	36.4 (196)	66.3 (293)	51.5 (50)	.006
No	63.6 (343)	33.7 (149)	48.5 (47)	

*Chi-squared test

At the start of the study (baseline t_0_), 6.9% (n = 37) of participants were edentulous and 93.1% (n = 502) had teeth (1–28 teeth). The deficits presented in edentulous persons were xerostomia in 16.2% and need for dental prosthesis (absence of stability and retention) in 70.3%. Of the dentate (< 20 functional teeth) older adults (52.8%), 28.5% had coronal caries (≥ 2 teeth), 19.0% root caries, 88.2% periodontal disease, 22.1% dental calculus, 26.1%, dental biofilm, 15.3%, in root remains, 13.5%, xerostomia, and 29.7% needed a dental prosthesis. Following the criteria for latent class formation, the decision was made to form three classes to categorize oral health from an entropy of .796, an Akaike Information Criterion (AIC) of 4254.034, a Bayesian Information Criterion (BIC) of 4376.373, and a Lo-Mendell-Rubin adjusted likelihood ratio test of *p* = .0394 (Table 3).

**Table 3:** Goodness of fit of the latent class model and conditional probability associated with oral health

Number of latent classes	AIC	BIC	Entropy	LMR test *p*-value
1	4711.440	4749.407	–	–
2	4288.799	4368.952	.805	.0000
3	4254.034	4376.373	.796	.0394
4	4239.008	4403.533	.715	.0954

Oral health deficits	Edentulous 6.9% (n = 37)	Class 3 Poor oral health 21.3% (n = 115)	Class 2 Somewhat acceptable oral health 13.9% (n = 75)	Class 1 Acceptable oral health 57.9% (n = 312)
< 20 functional teeth	100.0 (37)	.939	1.000	.256
Coronal caries		.647	.204	.170
Root caries		.531	.160	.072
Periodontal disease		.970	.911	.843
Dental calculus		.674	.112	.079
Dental biofilm		.623	.198	.141
Root remains		.343	.319	.041
Xerostomia	16.2 (6)	.164	.239	.099
Need of dental prosthesis	70.3 (26)	.594	.948	.022

*AIC* Akaike Information Criterion, *BIC* Bayesian Information Criterion, *LMR* Lo-Mendell-Rubin likelihood ratio test

**Fig. 2:**
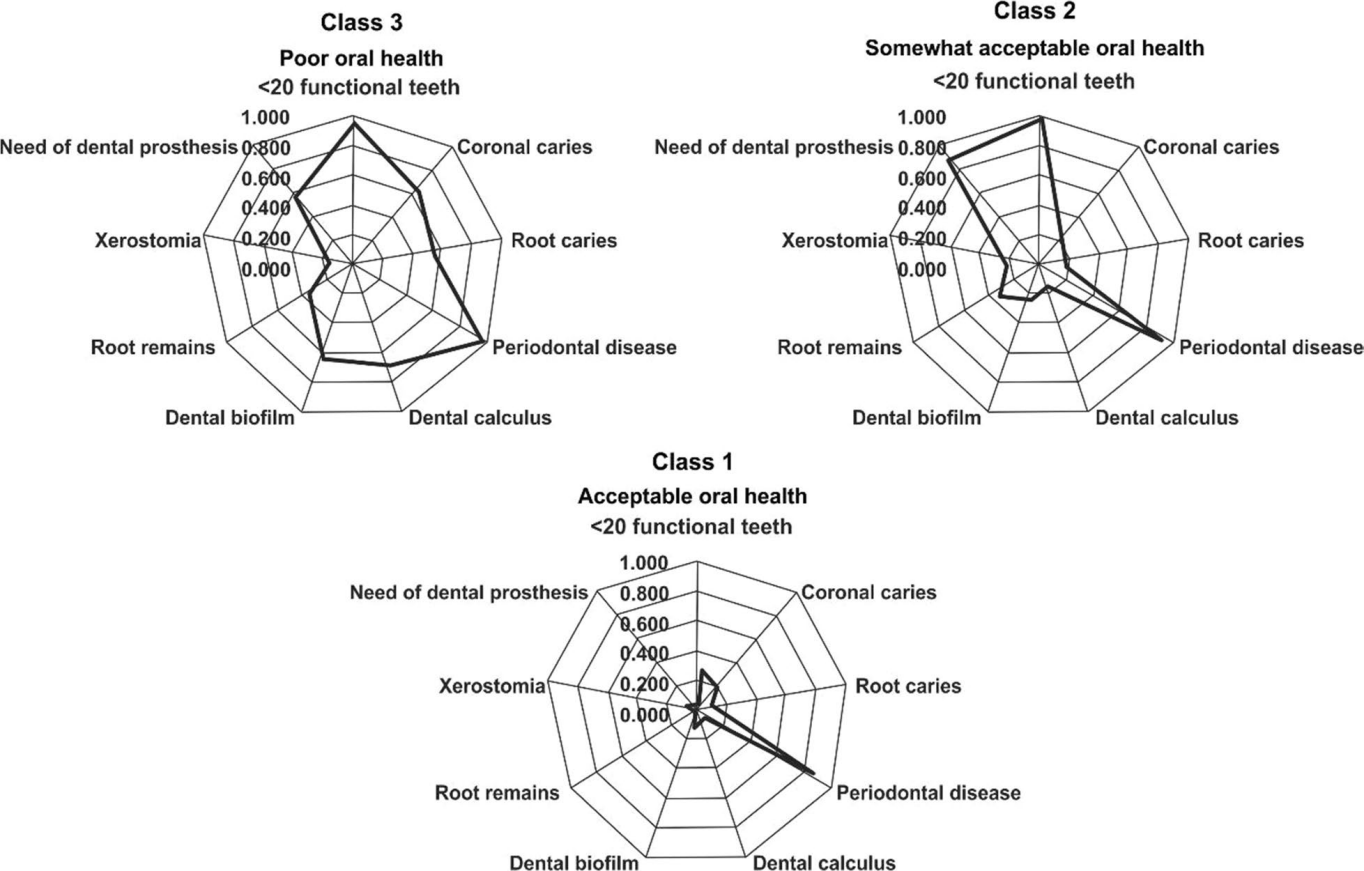
Latent class for oral health in older adults. OR adjusted for socio-demographic characteristics (sex, age, marital status, level of education, paid work activity, and living alone), comorbidities, cognitive impairment, depressive symptoms, nutritional status and use of oral health services

Figure 2 shows the probability of different oral health deficits in dentate older adults in each of the three oral health classes. Regarding the conditional probabilities of oral health deficits within each class, for Class 2, we observed a noticeable increase in the probability of < 20 functional teeth, periodontal disease, root remains, and the need for dental prosthesis compared with Class 1. Class 3 showed an increase in the probability of coronal caries, root caries, periodontal disease, dental calculus, dental biofilm, and root remains compared with Class 2. According to these probabilities, the categories were named as follows: Acceptable oral health (Class 1); Somewhat acceptable oral health (Class 2); and Poor oral health (Class 3). Finally, the following four categories were obtained: Edentulous older adults 6.9% (n = 37); Acceptable oral health (Class 1) 57.9% (n = 312); Somewhat acceptable oral health (Class 2) 13.9% (n = 75); and Poor oral health (Class 3) 21.3.% (n = 115).

**Fig. 3:**
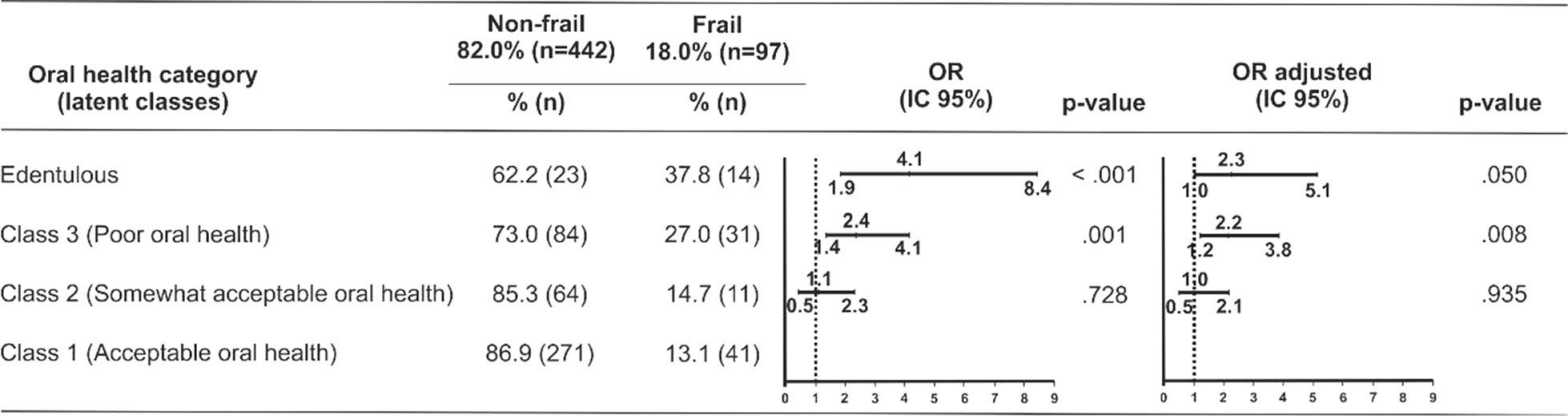
Strength of the association (OR) between frailty and the oral health classes in older adults

Using the oral health categories at baseline (t_0_), the strength of the association was estimated from the bivariate logistic regression analysis for the development of frailty at 12-months of follow-up (t_1_). The results showed that edentulous older adults and those with poor oral health were 4.1 times (95% CI 1.9–8.4) and 2.4 times (95% CI 1.4–4.1), respectively, more likely to become frail than older adults with acceptable oral health (Class I). The fully adjusted model showed that edentulous older adults (OR = 2.4; 95% CI 1.0–5.1) and older adults with poor oral health (OR = 2.2; 95% CI 1.2–3.8) were more likely to become frail than older adults with acceptable oral health. No association was noted for older adults with somewhat acceptable oral health compared to those with acceptable oral health (Fig. 3).

## Discussion

For this study, we determined the strength of the association (OR) of the oral health categories at baseline (t_0_) and development of frailty over a 12-month period (t_1_) in older adults. The bivariate analysis found that the strength of the association is greater in edentulous older adults and those with poor oral health. After adjustment for the other variables under study, the association between edentulism, poor oral health, and developing frailty in that period remained.

In total, 18.0% (n = 97) of the older adults included in this study developed frailty over a period of 12-months. This incidence rate was higher than those reported by Castrejón et al. and Ramsay et al. (14.8% and 10.0%, respectively) in older adults, in their respective three-year follow-up studies. Likewise, the incidence rate in our study surpassed that found by Iwasaki et al. (14.9%) in their five-year follow-up study in adults over age 75, as well as the overall incidence reported by Ofori-Asenso (13.6%) in the meta-analysis of 46 studies with a median follow-up of three years in populations aged 60 years and over [14, 18, 34, 35].

Comparing this study with one conducted by Castrejón et al., there is a difference in the development of frailty of 3.2% (14.8% cumulative incidence of frailty). They also used frailty phenotype, but unlike this work, they evaluated unintentional weight loss of 5 kg in the last 6 months, the slowness of gait, and grip strength, which is evaluated subjectively. Therefore, there could have been a reporting bias and probably an underestimation of the results due to the subjectivity of the aforementioned frailty criteria. In addition, the population of this study are residents of a district that is part of a Mexico City government program, so it is possible that they are under constant review and receive some type of care [14].

The difference in the development of frailty in this study compared with Ramsay et al. is 8 points. Although they also used the frailty phenotype, this difference may be due to the fact that they evaluated grip strength subjectively and slow walking speed was determined by self-reporting of usual running rate. In addition, unintentional weight loss was ascertained by self-reporting of weight loss in the past 4 years. Therefore, there may have been an information bias and possible underestimation because of the subjectivity of the assessment regarding the above-mentioned frailty criteria [18, 36, 37].

According to the LCA, a methodology to determine oral health typology, we considered three classes of oral health related to the studied parameters [6]. Doing so, we observed a stronger association between older adults with poor oral health and development of frailty over a 12-month period. Consequently, the LCA can be considered a useful tool for determining typology in other studies that need to classify older adults according to their oral health status [38].

Oral health ailments begin at early ages; without proper care, they can evolve into major problems at advanced ages. For example, tooth loss, edentulism, loss of clinical insertion, coronal and root caries, use of non-functional dental prosthesis (partial or total dentures), and chewing problems, among other conditions [9].

Oral health is generally evaluated in terms of deficits and indices, which does not provide a complete measure of oral health, like the patterns in this study [10]. Using latent class analysis for oral health conditions, we obtained a model of three classes in addition to the group of edentulous older adults. This is consistent with both Sánchez et al. and Ortiz et al., who also identified three classes in addition to edentulous participants, in a population covered by the social security system. It should be mentioned that, although the oral health deficits used to perform the latent class analysis were not the same among the aforementioned studies, similarities were observed so far as the highest percentage of the population was classified with favorable oral health [9, 10].

Regarding edentulism and dental loss, several reports [15, 16, 34] have identified tooth loss as a possible early indicator of frailty [39–41]. Dental loss, edentulism, and absent or inadequate dental prosthetic rehabilitations in older adults can affect several factors, such as nutrition [42–45], socialization, and quality of life [46, 47], which could probably lead to the development of frailty. Studies are needed to clarify the relationship between edentulism and tooth loss with the components of the frailty phenotype.

On the other hand, although a high probability of periodontal disease is observed among people with acceptable oral health, a high probability is not observed for other deficits. The probability of periodontal disease is higher among people who have more teeth; tooth loss cancels that probability. Also, edentulous people probably lost their teeth to periodontal disease, but this cannot be accurately determined. As for poor oral health condition, periodontal disease represents an important component.

Periodontal disease does not begin at an early age. It is an inflammatory process of periodontal tissues that is exacerbated when the disease is active. This can trigger higher levels of inflammatory markers, damage to tissue, or pathogenic bacteria can cause the gingival epithelium to initiate the inflammatory response in which epithelial cells release pro-inflammatory mediators such as macrophages, mast cells and polymorphonuclear cells, with secretion of interleukins IL-1 IL-6, IL-8 and tumor necrosis factor alpha (TNF-α) and histamine, which amplify inflammation and could contribute to the development of frailty [48, 49].

Among the limitations of the study is the uneven distribution of participants in the different classes. It would thus be necessary to increase the size of the sample or implement other designs in which the number of participants in each group remains fixed. Another limitation is the loss to follow-up of some participants (18.7%). The main reasons were not being located (48.4%), followed by not agreeing to be interviewed or examined (31.5%), which could have occurred because of illness or frailty. The literature has reported that the loss of participants during follow-up is associated with data missing not at random, so biased estimations may occur [50]. For these reasons, we suspect that the incidence of frailty could have been underestimated. Furthermore, being a community of older adults with social security coverage, the results cannot be generalized to the entire population of that age group.

An additional limitation was the evaluation of periodontal disease, which was defined as presence of clinical attachment loss ≥ 4 mm in at least one tooth. This measurement of periodontal disease generally considers people with at least moderate severity [51]. Therefore, a large number of individuals were classified as having the disease (88.2%) without the possibility of identifying those with severe periodontal disease, who may have had the highest risk of frailty because of the constant inflammatory process [5, 8]. Thus, the impact of each latent class could have modified the final result. It is necessary to continue studying the possible relationship between the inflammatory processes originated by periodontal disease and other diseases, in this case the development of frailty.

This study did not evaluate masticatory capacity with and without the use of prostheses. Therefore, other studies should consider it to determine if it has any implication in the development of frailty in older adults.

Hakeem et al. [16] and Tórres et al. [52] conducted literature reviews of studies with both cross-sectional and longitudinal designs, in which they report the association between clinically assessed oral health deficits and perception of oral health with frailty or any of its components in older adults. The number of teeth is among the main oral conditions associated with frailty, which is related to chewing problems and a need for rehabilitation with dental prostheses. This could limit food selection and processing, impacting nutritional status, which is one of the most recognized factors associated with the development of frailty. This could be a possible mechanism underlying the relationship between oral health and frailty [53]. There is a need for further research that explores the role of nutrition as a mediator between oral health and frailty.

With respect to the strengths of the study, one is its case-cohort design, in which cases develop according to the same chronological sequence of the case–control design nested in a cohort. The difference between these two designs is that controls in the case-cohort design are selected from the cohort with which the study began [20]. A longitudinal study can help establish a causal relationship between oral health and development of frailty over a short period of time. More years of follow-up would certainly show a greater incidence of frailty and the effect would remain significant in the fit analysis. Finally, using the frailty phenotype for the evaluation of the event of interest allows comparison with other studies. Yet, as far as we know, this is the first study to use a comprehensive approach to oral health and examine its relationship with the development of frailty.

Edentulism and poor oral health can be indicators of the presence or development of frailty, which in turn is considered as a potential public health problem in the older adult population [54, 55], given its association with adverse health outcomes such as falls, impaired mobility, functional dependence, disability, hospitalization, and institutionalization [5, 21].

Further research will be needed to confirm the association between oral health and the development of frailty in older adults, using longitudinal studies with other populations, such as those who are institutionalized or do not have social security. In this way, the causal relationships of highly preventable diseases can be explored.

Lastly, changes in operational definitions of oral health deficits may probably modify the distribution of groups, so it would be advisable to hypothesize using different operational definitions or different cutoff points to evaluate behavior when predicting frailty.

## Conclusion

The results of this study suggest that older adults with edentulous and poor oral health have an increased risk of developing frailty over a 12-month period. Given that oral health deficits can be prevented at all stages of life, intervention programs should be implemented to improve this population’s oral health.

## Data Availability

Data is available upon request. Contact e-mail: sergio.sanchezga@imss.gob.mx.

## Abbreviations

AIC: Akaike Information Criterion
BIC: Bayesian Information Criterion
CESD-R: Center for Epidemiologic Studies Depression Scale-Revised
COSFOMA: Cohort of Obesity, Sarcopenia, and Frailty of Older Mexican Adults
IMSS: Mexican Social Security Institute
LCA: Latent Class Analysis
LMR: Lo-Mendell-Rubin
MMSE: Mini-Mental State Examination
MNA: Mini Nutritional Assessment
OR: Odds Ratio
SD: Standard Deviation
